# Progress in Extrusion-Based Food Printing Technology for Enhanced Printability and Printing Efficiency of Typical Personalized Foods: A Review

**DOI:** 10.3390/foods11244111

**Published:** 2022-12-19

**Authors:** Xiuxiu Teng, Chunli Li, Arun S. Mujumdar, Min Zhang

**Affiliations:** 1 State Key Laboratory of Food Science and Technology, School of Food Science and Technology, Jiangnan University, Wuxi 214122, China; 2 Jiangsu Province International Joint Laboratory on Fresh Food Smart Processing and Quality Monitoring, Jiangnan University, Wuxi 214122, China; 3 Editorial Department of Journal of Food and Biotechnology, Jiangnan University, No. 1800, Lihu Avenue, Wuxi 214122, China; 4 Department of Bioresource Engineering, Macdonald College, McGill University, Sainte-Anne-de-Bellevue, QC H9X 3V9, Canada

**Keywords:** food printing, printability, printing efficiency, personalized foods

## Abstract

Three-dimensional printing technology enables the personalization and on-demand production of edible products of individual specifications. Four-dimensional printing technology expands the application scope of 3D printing technology, which controllably changes the quality attributes of 3D printing products over time. The concept of 5D/6D printing technology is also gradually developing in the food field. However, the functional value of food printing technology remains largely unrealized on a commercial scale due to limitations of printability and printing efficiency. This review focuses on recent developments in breaking through these barriers. The key factors and improvement methods ranging from ink properties and printer design required for successful printing of personalized foods (including easy-to-swallow foods, specially shaped foods, and foods with controlled release of functional ingredients) are identified and discussed. Novel evaluation methods for printability and printing precision are outlined. Furthermore, the design of printing equipment to increase printing efficiency is discussed along with some suggestions for cost-effective commercial printing.

## 1. Introduction

The existence of people with special food needs (for example, children, the elderly, vegetarians, pregnant women, and patients) has stimulated the development of innovative methods to design and create personalized foods [1]. Personalized foods include foods with many characteristics, such as taste, texture, color, shape, and nutrition [2]. Food printing technology can reshape food materials through layer-by-layer deposition based on pre-designed models, which is a potential technology to manufacture personalized foods [3].

Food printing technology displays many advantages such as the ability to produce personalized food customization, digital nutrition matching, simplified supply chain, reuse of food scraps, and widening the source of food ingredients [4]. These advantages have prompted food printing technology to receive extensive attention from different countries since 2012. Companies include, for example, Redefine meat, Aleph farms, Meat-tech 3D from Israel, Cellx, Moodles from China, and Modern meadow from the United States. The latter are engaged in 3D printing of meat, Shiyin technology from China is committed to the research of printing chocolate, Open Meals from Japan opened the first “3D printing sushi” restaurant, and 3D systems from the United States has launched a 3D printed candy [4]. The market for 3D food printing was valued at $34.7 million in 2019 and is projected to exceed $1 billion by 2027 [5].

In terms of the modes of food printing, extrusion-based printing is the most widely used technique in the food sector [6]. Extrusion-based printing includes hot-melt extrusion and room-temperature extrusion. Hot-melt extrusion depends on the higher temperature of the printing nozzles to change solid raw materials into a liquid state for extrusion, and the extruded material solidifies on the printing platform due to the temperature difference. Room-temperature extrusion does not experience temperature changes, and it achieves printing directly through the stacking layers of lines [7]. The most representative food material that needs hot-melt extrusion is chocolate, followed by some hydrogels such as carrageenan and agar, in addition to meat, starch, vegetables, and fruits. Notably, some hydrogels have been successfully printed through room-temperature extrusion [8].

Based on extrusion-based printing technology, various personalized foods have been produced successfully to meet the unique needs of consumers. However, society requires innovative technology to not only maintain or enhance personalized value, but also to be sustainable and competitive. There are difficulties in scaling up personalized foods from the current level to industrial production. Printing accuracy and printing speed are major concerns for food printing technology. Several recent review articles published on food printing technology have focused on the impact of ink characteristics, printing parameters, and post-processing on printing formability at the household level [9,10,11]. The article published by Le-Bail et al. [12] described the concept of extrusion-based printing, the types of extrusion-based printing, the basic requirements of printing inks in a very short space, and described the limitations of large-scale production of 3D printing technology. This review differs from and supplements currently available reviews in a number of features. These differences are reflected in the following aspects. (1) The factors and improvement methods that realize accurate and fast printing of three typical personalized foods including easy-to-swallowed foods, specially-shaped foods, and foods with controlled release of functional ingredients are discussed. (2) Novel methods to evaluate the printability of raw materials are identified. (3) The design of printing equipment is outlined so as to achieve mass production of personalized foods.

## 2. Printability of Three Typical Personalized Foods

Printability is defined as the similarity between the printed object and the printing model, and the sustainability of the printed object in the original structure and function during post-placement or post-processing [7]. Printability depends on the characteristics of printing inks and the printing ability of the specific printing system. Next, to improve printability, the characteristics of printing inks and printing systems for three typical personalized foods, including easy-to-swallow foods, specially-shaped foods, and foods with controlled release of functional ingredients, are discussed.

### 2.1. Characteristics of Printing Inks

Printability of printing inks is mainly reflected in rheological properties (for example, yield stress, viscosity, shear-thinning behavior, storage modulus, and shear recovery) during the printing process and shape stability during the deposition process [13,14]. Printing inks should behave as thixotropic fluid that can be easily extruded but very quickly increase its viscosity to eliminate flow out of printed products.

#### 2.1.1. Easy-to-Swallow Foods

Dysphagia refers to the impossibility of swallowing liquid, semisolid, or solid food. This condition affects almost 580 million people worldwide, especially infants and the elderly, and it leads to nutritional deficiencies [15]. The demand of dysphagic patients with respect to their diet is both safe replenishment of water and nutrition, while maintaining food enjoyment for quality of life [16]. However, dysphagic patients tend to eat pasty, moist, and liquid foods that are visually unappealing. Three-dimensional printing technology provides a continent solution to standardize and automate the preparation of muddy foods with repeatability and visually attractive appearance [17]. The suitability for dysphagic patients of printed samples was evaluated in terms of texture properties (for example, hardness, cohesiveness, and gumminess) and International Dysphagia Diet Standardization Initiative (IDDSI) recommended tests. For example, Du et al. [18] used a mixture of rice, flour, yeast, and water as the printing ink to prepare the easy-to-swallow steamed buns. Pant et al. [16] and Xing et al. [19] used hydrocolloids (for example, xanthan gum, kappa carrageenan, and locust bean gum) to modify the printability of freshly pureed vegetables and successfully provide the printed foods to dysphagic patients. Dick et al. [20] and Chao et al. [21] used 3D printing technology to design delicious and soft meat products through the internal structure design. Printing inks used to prepare easy-to-swallow foods usually require high water content and a weak network structure. Starch, protein, or hydrocolloids are the main auxiliary materials to enhance the printability of raw materials. For fruits and vegetables with high water content and low starch/protein content, hydrocolloids such as xanthan gum, carrageenan, and Arabic gum are usually added to improve extrudability, printing accuracy, and shape retention because the addition of hydrocolloids is less, and the texture of printed products is softer and is finer than that mixed with starch/protein [22,23]. In addition, compared with starch/protein, the addition of hydrocolloids can better increase the stability and rheological properties of food foam, thus reducing foam rupture during the extrusion process and increasing the printability of printing inks. More foam structure allows the printed food to stay in the mouth for sufficient time to provide hydration while reducing the danger of choking [24].

The combination of ions and hydrocolloids (for example, calcium ions and low-methoxyl pectin/alginate/gelatin) is found to further improve printing accuracy and reduce the degree of collapse because ions enhance the network structure of hydrocolloids. Negatively charged carboxylic acid groups in hydrocolloids combine with positively charged Ca^2+^ ions, which are then surrounded by galacturonic residues to form junction zones leading to a gel network [25].

**Figure 1:**
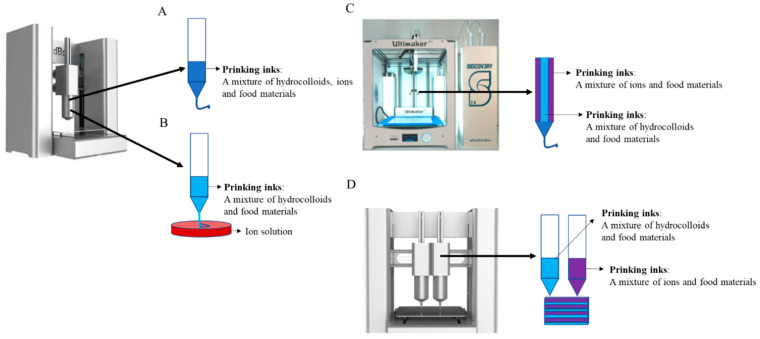
Combination mode between ions and hydrocolloids in the 3D printing process to enhance the printability of raw food materials (**A**) single nozzle printing with the mixed materials (**B**) single nozzle printing with the separated material (**C**) single nozzle printing with a coaxial nozzle (**D**) printing with double nozzles (arrow meaning where the nozzle is placed) (Reprinted with permission from Vancauwenberghe et al., 2018 [26]. © 2018, Elsevier Ltd., Amsterdam, The Netherlands) [26].For fruits and vegetables rich in high starch/protein content, these food materials have a good self-supporting ability, but they often break lines in the process of printing due to the rigid network structure. One solution is to add lubricants such as fat, phospholipids, or small molecules such as maltose and catechin to increase the softness of the network structure. Teng et al. [27] found that the addition of oils changed the rheological properties of *Cordyceps* flower powder and reduced the phenomenon of line breakage. Zeng et al. [28] found that catechin or procyanidin was attributed to improving the printability of starch-based materials due to the enhanced softness of the lines. Another solution is to use the physical field (for example, ultrasound, microwave, and radio frequency) or chemical methods to pretreat the raw materials in order to change the rheological characteristics and network characteristics of the materials. Xu et al. [29] found that ultrasonic treatment could destroy the structure of the wheat starch-papaya system, reducing viscosity and hardness, while increasing the ratio of bound water and improving the ability to retain its shape. Maniglia et al. [30] found that ozone-modified cassava starch increased the number of carbonyl groups and carboxyl groups, and the modified starch showed the lowest apparent viscosity, improved gel texture and good printability at all temperatures.

As shown in Figure 1, ions can be added in four ways to enhance the network structure of hydrocolloids [26]. (1) Ions and hydrocolloids are added to the raw materials together and printed by the single nozzle (Figure 1A). (2) The hydrocolloid is firstly mixed with the raw material and printed through the single-nozzle printer, and the deposited lines are fed into an ionic solution for solidification (Figure 1B). (3) With the help of the single-nozzle printer with multiple channels, one channel contains hydrocolloid-raw material mixtures, and the other channel contains ion-raw material mixtures. In the printing process, the materials of the two channels are extruded at the same time and meet at the nozzle, forming a deposition line (Figure 1C). (4) With the help of the double-nozzle printer, hydrocolloid-raw material mixtures and ion-raw material mixtures are added in different nozzles. The two nozzles are printing alternately in order to enhance the mechanical strength of the line contact interface (Figure 1D). These four methods described above allow printed products to exhibit different physical properties. For example, method (1)/(2) possessed the higher printing accuracy, followed by methods (3) and (4). The shape retention ability and the hardness of printed products is ranked from high to low as in methods (1), (2), (3), and (4). On this basis, the addition of physical fields is also able to enhance printability. Guo et al. [25] used microwave heating combined with Ca^2+^ to decrease the viscosity of the starch-pectin mixture while to improve its resistance to deformation, thus enhancing the printing performance. In real life, the printing method and the hydrocolloid species should be selected according to the degree of dysphagia.

For protein-rich liquid foods like dairy products, in addition to adding hydrocolloids, a gel of protein itself under the action of enzymes is also a common way [31]. Two types of milk proteins (casein and whey) can form a gel. Casein gel is a well-studied topic because it is an essential process for preparing cheese. Casein gels can be formed by selective hydrolysis of micelles by aspartic protease, acidification to isoelectric point values (4.6), or a combination of both [32]. The process of gel of casein by aspartyl protease includes hydrolysis of κ-casein and aggregation of micelles [33,34]. Protein content, temperature, ionic strength, pH value, and enzyme concentration can affect the gel time and the strength of the obtained gel, thus leading to differences in printability and ease of swallowing [35]. In conclusion, printing technology is feasible to produce easy-to-swallow foods through formulation adjustment and internal structure design [36]. In the process of preparing the printing formula, the additives should be reduced as much as possible under the condition of successful printing. At the same time, a lower filling ratio can provide softer texture [37].

#### 2.1.2. Specially Shaped Foods

Specially shaped foods enhance the interaction between diners and food materials. According to the path planned by slicing software, food printing technology successfully prints a variety of food shapes by layer-by-layer deposition. Although 3D printing technology can produce some shapes that are difficult to be replicated by molds, it is still difficult to print perfectly in shapes with few support points such as suspension, torsion, tilt, and hollow structures. Four-dimensional printing technology is an extension of 3D printing technology. In other words, 3D-printed products undergo controllable changes in sensory qualities (for example, shape, and color) or nutritional qualities under external stimuli such as heat, pH, and light. Four-dimensional printing technology opens up ideas for the production of specially-shaped foods. Besides attracting consumers, specially shaped foods induced by 4D printing technology allow consumers to judge the cooking degree of foods according to sensory changes. It also fits well with the concept of “flat packaging”, which converts foods from 2D to 3D after processing, thus reducing packing costs and storage space [4]. In the field of food, the stimulus factors leading to deformation mainly include water absorption and dehydration, and the main components of printing inks contain starches, hydrocolloids, and celluloses. The degree and direction of deformation caused by 4D printing technology depend on the nature of raw materials, the spatial arrangement of different ingredients, and the groove design on the product’s surface. The expansion difference caused by water absorption of food materials is one of the reasons for inducing deformation. Printed structures are composed of expandable hydrophilic materials and rigid materials [38]. According to the above anisotropic expansion differences, the collocation of gelatin and ethyl cellulose has been used to prepare films that curl when exposed to water. Among them, gelatin can expand obviously after encountering water, and the ethyl cellulose can control and adjust the degree of deformation through cellulose density, thickness, line gap, and total coverage. The film has been used in food applications such as corn crackers, water sushi, and self-packing cannoli. The precondition for the success of this deformation is that the two materials are closely bonded, otherwise, the reconstituted structure is easy to separate in the water environment.

Dehydration stimulation is also one of the common methods to induce the deformation of printing products. The difference in the dehydration rate is the main factor causing product deformation. The main factors causing variation in dehydration rate include the heating method and product formulation. Liu et al. [39] investigated the effect of different drying methods on the degree of deformation and found that traditional hot-air heating resulted in a larger bending angle, while the sample treated by the microwave and infrared heating had smaller deformation angles due to surface hardening induced by the rapid evaporation of surface water. He et al. [40] found that the salt or sugar content of the printing formula could affect the heat transfer process induced by microwaves and thus influence the deformation of the sample. Improper evaporation of water can lead to cracks or voids in the printed product. In addition, the internal design of the printed sample, such as filling the shape, filling angle, and filling ratio, is also the influencing factor that causes the transformation of the printed product from two to three dimensions (as shown in Table 1). He et al. [40] found that the bending angle of the printed product decreased with the increase of the filling angle. Additionally, Teng et al. [36] found that the high filling density prevented water vapor from escaping, resulting in an undesirable expansion of the sample. Guo et al. [41] used the local expansion caused by the local hot spots generated by the groove to successfully realize the blooming of the bud and Tao et al. [4] successfully prepared a variety of curling structures using this groove design and applied them to food fields such as pasta and snacks.

The 5D printing concept is a recent technological innovation, which includes the movement of five axes, namely three axes of movement of the print head and two rotational axes of the print bed [43]. Compared with 4D printing, 5D printing is used to create difficult and intricate shaped products and could produce the most complex and curved structure with less material, with high potential applicability [44]. Currently, 5D printing has been successfully used in healthcare and automotive applications, where high-strength bending and complex shapes are required [43]. In terms of 5D printing, the rheological properties of printing inks were a prerequisite requirement for printing concave shapes or curved layers from various dimensions. However, the rheological properties of edible materials are difficult to achieve accurate 5D printing, compared to synthetic polymers. In addition, 6D printing products are considered to be 5D printing products that undergo controlled sensory changes under external stimuli. Six-dimensional printing combines the high-strength structure of 5D printing and the shape, color, and taste changes over time in response to the stimulation from 4D printing, thus helping to prepare more creative foods and to expand the range of services for specific groups [45]. Therefore, 6D printing inks must be responsive to external stimuli on the basis of meeting 5D printing requirements. Currently, 6D printing has not been used in any field. To advance the development of the food field, 5D/6D printing requires further creation and the combination of various fields such as rheology, mathematics, engineering, and biology.

#### 2.1.3. Controlled Release/Conversion of Functional Ingredients

Compared to model-built products, 3D printing technology can influence changes in release rates while providing a higher degree of flexibility in dose size, shape, and appearance. Kamlow et al. [46] compared the differences in the release of cinnamaldehyde and erioglaucine disodium salt by carrageenan emulsion gel prepared by 3D printing technology and a cylindrical mold. Compared with this mold, 3D printing technology enabled the co-release of hydrophilic and lipophilic substances. Additionally, some sensory qualities or nutritional qualities can gradually decline with the extension of storage time. Four-dimensional printing technology can realize the release of these qualities of the printed products when receiving external stimuli, which greatly preserves the edible value of products. For example, Phuhongsung et al. [47] used spray solutions with different pH values to induce flavor changes in 3D-printed products containing pumpkin, beetroot powder, and soy protein isolate. The results from the electronic nose showed that aromatic compounds and terpene esters increased at pH 8 and 10. While using a microwave (50 ~ 80 W, 20 min) to stimulate 3D printed products containing carrageenan, vanilla, and soy protein isolate for producing shape changes, four new flavor compounds (1-Octen-3-ol, maltol, ethyl maltol, and eugenol) were released in addition to increases in umami and salty flavor characteristics [48]. Chen et al. [49] used ultraviolet stimulation to convert ergosterol into vitamin D2 in mushroom chips and found that the concentration of vitamin D2 in the printing model was 4.6 times higher than that of direct irradiation of raw materials.

Besides adding the released material directly to the printing ink used as a carrier, the released material can also be encapsulated in microcapsules and then added to the carrier. Compared with the method of direct addition to the hydrogel, microcapsules are more conducive to protecting functional substances. However, the development of microcapsules with a specific response to external stimuli is not perfect, and the migration rate of released substances in the carrier is slow, which lowers the product output. Currently, gelatin-Arabic gum composite coacervate is considered a potential wall material that can be used for 4D printing because of it is a thermo-responsive material [25]. In addition to the rupture of microcapsules under special stimulation, it should be noted that the wall materials of microcapsules need to be able to maintain integrity in the extrusion process, and the concentration of released substances can meet the requirements of discoloration, deformation, or nutrition supplement [50].

In conclusion, food printing technology provides a new idea for the controlled release of functional substances through the structural design of printing models in the porosity, the contact area with the stimulus source, and the spatial distribution of functional substances.

### 2.2. Printing Ability of Printing System

The printing system mainly includes the mechanical part for implementing printing and the software part for controlling the printing path. The mechanical part mainly includes the extrusion device, transmission device, printing platform, and some auxiliary [25]. Among them, the extrusion device is divided into piston type, atmospheric pressure, and rotary screw. Piston type and atmospheric pressure depend on the pressure difference to extrude food inks. For the screw extrusion, food inks are mainly distributed through the rotary screw and the speed of the motor determines the distribution amount [51]. Compared with screw extrusion, the biggest defect of the extrusion device based on pressure is that when the pressure disappears, the flow of food inks cannot be stopped immediately because of inertia, leading to too much material being extruded, thus reducing the printing accuracy. In addition, screw type conveys materials as more stable than pressure type, which is important for printing accuracy [52]. For screw type printers, there is a gap and high shear between the extrusion pipe wall and the screw, leading to a risk of backflow in the process of printing foods of high viscosity.

The Cartesian coordinate system is the traditional mode used in 3D/4D printing equipment due to its simple structure and easy manufacture. Common Cartesian coordinate systems involve movements in *X*, *Y*, and *Z* directions, and the movement modes of the printing head and printing platform can be summarized into three categories [4]. (1) The printing head moves along the *X*-axis and *Y*-axis directions, and the printing platform moves independently along the *Z*-axis direction. This form is suitable for printing a compact model; however, the normal discharge of the nozzle can be affected by the composite movement of the nozzle in the *X*-*Y* plane, thus reducing the printing quality. (2) The printing platform moves in the direction of the *X*-axis and the *Y*-axis, and the printing head moves independently in the direction of the *Z*-axis. This form requires precise control of *Z*-axis motion, high mass of the worktable, and lower printing speed. (3) The printing head/printing platform does composite motion in the direction of the *Z*-axis and *X*-axis (or *Y*-axis), which has small movement inertia and the overall structure is simple. This composite motion is widely used in the structural design of household 3D printing equipment. In order to include a variety of food materials in one printed object, the two-nozzle printer was developed [9]. Compared with the single-nozzle printer, the most critical factor affecting the printing accuracy of the two-nozzle printer is the precise positioning of the nozzle when the nozzle works alternately. Besides the three axes (*X*, *Y*, and *Z*) of movement for the printing head used in 3D printing technologies, the five axes of 5D printing result from the two extra movements of the printing platform on two rotational axes [43]. High-precision printing requires that the five axes are matched with each other and positioned accurately. The precision required by 5D printing equipment and the two additional shafts increases manufacturing cost, which also limits application in the food field [44].

The software part (mainly referring to modeling software, slicing software, and system control software) is the command center controlling the mechanical part. Specifically, modeling software such as 123D, AutoCAD, and Rhinoceros are templates for printing [6]. In order to print accurately 3D printing models, some details should be noted [15,53]. (1) The number of suspended structures should be minimized during the design of 3D printing models, and the angle between each part of 3D printing models and the horizontal plane should be higher than 45° in order to prevent collapse. For reducing the collapse phenomenon, support points can be added. However, the addition of support points will consume more material and damage the appearance. (2) The thickness of 3D printing models should be as large as possible to avoid errors during slicing. The minimum thickness is usually 2 mm, depending on the type of 3D printer. (3) Three-dimensional printing models must be a whole and the parts of the model are continuous, which can be checked using some software such as the STL check function of 3Ds Max, the automatic boundary detection function of Meshmixer, and the repair function of Magics software and Netfabb software. (4) The size of 3D printing models should be less than that of the 3D printer. When the model size is larger than the printer’s capability the model color will appear gray, or the printer will have an error message. (5) The printing base of 3D printing models should be flat in order to improve the stability of the numerical model.

The main role of slicing software is to split 3D models into 2D models and generate G codes based on the layering path, required speed, and amount of extrusion [54]. Slic3r, Kissslicer, and Simplify are common slice software due to their faster slice speed and better slice quality [55].

**Figure 2:**
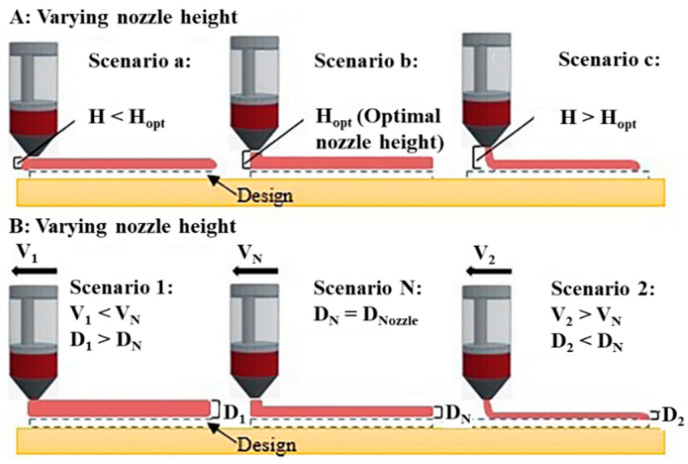
Effect of different nozzle heights (**A**) and different printing speeds (**B**) on streamline accuracy (Reprinted with permission from Dick et al., [20]. © 2019, Elsevier Ltd., Amsterdam, The Netherlands) [20].

The control system software can influence the accuracy of printed products through nozzle diameter, printing height of single-layer, printing speed, extrusion rate, filling rate, and filling pattern, alone or in combination [20]. A nozzle diameter deemed too large (more than 2 mm) is helpful to extrude food inks containing large particles, but it often affects the printing accuracy due to thicker lines. Relatively speaking, a smaller nozzle diameter (less than 2 mm) can improve the printing accuracy, but fine particles of print inks are required to prevent nozzle clogging. The printing height of single layer is usually equal to the nozzle diameter. When it is higher than the optimal value, there will be a gap between the nozzle and the printed product, resulting in dislocation and dragging between layers in the printing process. When it is lower than the optimal value, the nozzle will be inserted into the printed product, resulting in an expanded object compared with the desired design (as shown in Figure 2A). If the nozzle speed and extrusion rate are not set properly, the phenomenon of dragging, underdeposition, or overdeposition of printing inks will be observed (as shown in Figure 2B). The filling ratio is closely related to the stability of the printed product. A high filling ratio means more deposited food material and smaller porosity. More deposited food materials inevitably increase the gravity of the printed product, so the printed product needs strong self-supporting ability to prevent collapse. The porosity affects the stability of the printed product during storage. For example, Teng et al. [36] found that porosity affected the heat transfer efficiency in the heating process, thus affecting the shape change of the printing product of mashed potato. Huang et al. [56] found that small porosity reduced the water loss of printing products and enhanced the storage stability of printing products. The setting of the filling pattern also affects the accuracy and stability of printed products. For example, the filling pattern of linear or honeycomb that can provide more adhesion points allows the internally filled food materials to combine with the vertical shell, thus enhancing the stability of the printed object, while the concentric circle pattern lacks adhesion points unless a filling rate of 80–100% is used.

## 3. Evaluation Methods of Printability

Sensory evaluation methodology is the most intuitive method to reflect printing accuracy. However, it cannot quantify the printing accuracy and cannot judge the internal situation of printed products [57]. Shape fidelity of printed products becomes a popular method in order to quantify printing accuracy [58]. Shape fidelity refers to the shape-matching degree between the printed product and the model. This degree of matching is quantified by the difference in angle, height, and width between the printed product and the model by taking pictures. Liu et al. [59] took pictures of printed products with a camera and then used ImageJ software to analyze the printing angle, height, and width of key components of the printed products in order to comprehensively judge the printability of food materials. Fahmy et al. [60] developed a method to assess the printing quality of starch-based materials by morphological recognition using two onboard cameras. In this study, two cameras captured views from different angles and recorded printout parameters, which can be used as a basis to evaluate the printing defects of food materials.

Formulation change can cause printability of printing inks to deviate from desired characteristics. Therefore, to save time, manpower, and material resources, it is important to have some means of evaluating the printability of food materials before printing. Table 2 shows various novel evaluation methods in use for printability of food inks. Currently, rheometer and texture analyzer are the important tools for evaluating the printing characteristics of food materials because the physicochemical properties of food materials, such as linear porosity, mechanical properties, and network structure, are crucial to printability [58]. For rheometer, elastic modulus and composite modulus reflect the elasticity and stiffness of the raw material, therefore, these rheological properties can be used as indicators of material support. The power-law models of the consistency index and the flow behavior index are widely used to reflect the viscosity and shear thinning characteristics of the raw materials [61]. The dynamic mechanical loss angle tangent (tan (δ)) helps to determine whether the material exhibits viscous or elastic behavior. A value of tan (δ) less than one indicates an elastic behavior, while a value higher than one indicates a viscous behavior [62]. Nijdam et al. [63] used storage modulus and a decay factor to draw the graph and defined a dimensional stability window on the map by setting limit change parameters. This window was used to quickly monitor the rheological characteristics of food materials, and then predicted the stability of 3D printed products. For the texture analyzer, the maximum peak value was taken as the bloom strength/rupture strength of gel properties [36].

In addition, mathematical model-based simulation and equipment applied to food fields such as drying and detection are gradually introduced into the field of food printing to achieve a faster and more accurate evaluation of the printability of printing inks [64].

The moisture content of printing inks can significantly affect the physical properties of food materials. The technical principle of low-field nuclear magnetic resonance (LF-NMR) was based on the spin-relaxation properties of hydrogen nuclei from water in a magnetic field [65]. LF-NMR technology can determine the moisture distribution and the moisture content, so as to indirectly reflect the physical properties of raw materials. Liu et al. [66] evaluated the printability of mashed potato using LF-NMR technology according to the following steps. (1) The rheological and printing properties of potato purees with different formulations were determined. (2) The accuracy of printability of food inks was evaluated by principal component analysis and Fisher discriminant analysis. (3) The correlation between water relaxation time and rheological properties was established. Sun et al. [67] used LF-NMR technology combined with Pearson analysis to evaluate the effect of microwave treatment on dough rheological properties and printability, and established a model for rapid prediction of dough printability with an accuracy of more than 90%. Based on the above research ideas, Phuhongsung et al. [68] and Chen et al. [69], respectively, verified that LF-NMR technology also had more than 90% accuracy in judging the printability of soy protein isolate and surimi. Beyond that, other instruments (for example, differential scanning calorimeter, Fourier transform infrared analysis, and wide-angle X-ray diffraction) are also used to predict printability because these instruments can determine some physicochemical properties related to the rheological properties of food inks. For example, Liu et al. [70] used Fourier transform infrared spectroscopy to analyze the degree of short-range order of potato starch and judged the rheological properties of starch through the degree of order. Chen et al. [71] developed a new alternative use of near-infrared spectra to indirectly predict the 3D printability of purple sweet potato sauce. The measurement procedure was consistent with that described above using LF-NMR technology as an assessment mean of printability. Ralph et al. [58] used medium-wave and long-wave infrared cameras to measure the temperature profile of thermoplastic food materials during extrusion, thereby monitoring and regulating the fluidity of food inks.

Mathematical modelling and simulation can be used to display the fluid characteristics of food inks in the extruder by solving the model equations [72]. Guo et al. [73] used mathematical simulation to compare the effects of screw extrusion and piston extrusion on the printability of food inks and the results were consistent with the actual printing results, suggestive of the potential of mathematical model simulation to predict the printability of food materials. In addition, Guo et al. [50] introduced a Bird-Carreau model to determine whether different gels were subjected to the appropriate pressure in the extrusion-based printing process, and they found that the simulated value was positively correlated with the extrudability of the material. Jonkers et al. [74] successfully evaluated the mechanical properties of printing materials by identifying the numerical model parameters using the finite element algorithm. Duty et al. [75] developed a material evaluation model based on rheological and thermal properties for various extrusion printing methods, and the results of model evaluation were consistent with the experimental results.

**Table 2:** Evaluation methods/techniques for printability of food inks.

Method/Technology	Determination of Indicators	Application Process	Material	Advantages	References
Rheometer	Storage modulus, loss modulus, consistency index, flow behavior index, dynamic mechanical loss angle tangent	Before printing	All food materials	The related indexes can directly reflect the rheological properties of food inks.	[58,63,70]
Texture analyzer	Bloom strength/rupture strength of gel properties	Before/after printing	Food materials in a solid state	It directly reflects hardness and flexibility of the gel network and the self-supporting ability of the printed object.	[36,61]
Low field nuclear magnetic resonance	Water content and water distribution	Before printing	Food materials without oils	The correlation between water content/water distribution and rheological properties is established, which simplifies the number of measurement indicators and saves measurement time.	[66,67,69]
Photo comparison	Differences between photo and model (height, width, surface smoothness, bending angle, etc.)	After printing	All food materials	This method can reflect the printability of food inks most intuitively and accurately.	[70,76]
Mathematical model simulation	Changes of rheological properties of printing inks in different force fields and temperature fields	Before printing	All food materials	It helps to find the factors that affect the printability of printing inks.	[25,72,75]
Spectrum (Fourier transform infrared analysis and wide-angle X-ray diffraction)	The structural changes of macromolecules forming the gel network, and the relationship between structural change and rheological properties	Before printing	Food materials rich in starch and protein	The advantages are similar to those of low field nuclear magnetic resonance technology.	[66,71]

## 4. Factors Affecting Printing Efficiency and Improving Methods

In addition to nutrition, color, taste, and texture as key elements of the consumption experience, the amount of food printing must be replicated to achieve commercial success. So far, the large-scale production of printed foods is still a future concept. Only increasing the speed and nozzle diameter of a printer will not only reduce the printing accuracy due to the presence of broken lines, line deformation, and expansion, but also fail to meet the requirements of commercial production. A potential approach involves using a multi-nozzle printer (number of nozzles ≥ 3) to make simultaneously multiple objects. The multi-nozzle printer referred to here has different designs according to single material printing and multiple material printing. For single material printing, the design of multiple-nozzle printers entails the connection of multiple single nozzles in series. Notably, the number of nozzles cannot be adjusted. After the design upgrade, the number of printing heads is modularized, and the number of printing heads can be added or reduced according to production needs. For multiple material printing, multiple groups of printing heads used for single material printing are added, and the added quantity of printing heads is consistent with the type of printed materials. The limitations of this idea are attributed to the following factors: lack of automatic feeding system, a limit on the number of printing heads, precise positioning of multiple printing heads, separation between printing and post-processing, automatic transmission system, slice quality of software, and quality monitoring, etc. [77].

**Figure 3:**
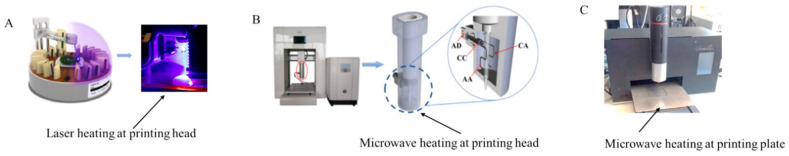
Examples of an existing integration device including printing and heating (**A**) laser heating (**B**) microwave heating at printing head (**C**) microwave heating at printing plate (Reprinted with permission from Hall et al., [80]. © 2021, Frontiers Ltd., Lausanne, The Switzerland.) [80].

For improved slice quality of software, the researchers propose to improve the print speed by adaptive algorithms, which can adjust the print parameters to balance print quality and time [78]. The problem to be solved by continuous feeding is that the material cannot be evenly distributed among multiple side-by-side printing heads. Compared with piston/atmospheric pressure extrusion, spiral extrusion has a higher printing accuracy and stability under the demand of rapid printing [12]. In order to facilitate maintenance and repair, the drive of the printing head is realized by pulling a belt. However, after the printer runs for a long time, the belt will become loose, resulting in inaccurate positioning [43]. Therefore, it is urgent to find a transmission mode or a material to replace the action of a belt. The printing process at the laboratory/household level is in a state of separation between the printing process and the post-processing process, which requires human participation. Printed products may be damaged and reduce production efficiency in the process of transportation [79]. To solve this problem, some researchers installed heating equipment such as a laser and microwave at the printing head to achieve real-time processing (as shown in Figure 3A,B). This design has been verified in print products such as salmon surimi and dough [4]. However, installing heating equipment at the printing head will increase the burden of the motor, increase the manufacturing cost of the equipment, and increase the risk of microwave radiation [80]. Another method is to install the heating device at the heating plate (as shown in Figure 3C), nevertheless, this will affect the heat transfer efficiency [70]. In order to increase production efficiency, our team is developing an infrared heating device that can automatically rise and fall. This device is installed on the printer frame to reduce the burden on the motor, increase heat transfer efficiency, and increase the product processing capacity. Quality monitoring of printed products can use photo comparison technology to monitor shape and color; electronic tongue or electronic nose to monitor flavor; and spectral technology to monitor product nutrients [76,81]. In actual industrial production, each production process can be connected through the transmission belt to achieve automatic production. In addition, bacteria will accumulate in the corners of the printer after long-term use, thus polluting printed products [82,83]. Therefore, we need to regularly replace needle tubes that are in direct contact with food inks or use safety coatings of food grade. In addition, the particles in the printer may migrate to the printed food, so printers need to use food-safety certified materials [82].

## 5. Suggestions for the Future

Three-dimensional food printing is a rapidly evolving technology, which makes it possible to produce innovative and unique products on demand. This technology is already well developed and applied commercially in many fields. For successful and cost-effective application in the food industry, much research and development are needed at the basic as well as the industrial scale to meet the special requirements unique to the food industry. The following recommendations are made on the basis of the relevant literature which is available.

(1) Modification methods need to be researched in depth to improve printability of diverse printing inks and for smart food materials suitable for 4D/6D printing.
(2) To minimize empirical trial and error tests, it is important to understand the fundamentals of the operation with appropriate numerical modeling. This is especially critical in view of the enormous variety of foods and their composition which affect their printing characteristics.
(3) A food-grade coating with antibacterial properties has to be developed in order to prevent bacteria from accumulating in the corners of the printer and printers also need to use food-safety certified materials in order to prevent metal particles from migrating into printing inks.
(4) Commercial utilization of 3D printing technology requires extensive testing for scale-up based on small-scale tests. Currently, printing technology can hardly compete with such food products that are produced by continuous processing lines, such as standard bakery products including buns rolls and bread. Automatic and intelligent printing equipment with multiple nozzles needs to be urgently developed.
(5) Quality standards for food safety, color, aroma, texture, and composition of nutrients need to be established for personalized foods such as easy-to-swallowed foods, specially-shaped foods, and foods with controllable release of functional substances.

## 6. Conclusions

Three-dimensional printing technology has great potential in manufacturing personalized foods. Four-dimensional printing technology adds a time dimension on the basis of 3D printing technology, which leads to controllable changes in the quality of printing products over time. In addition, 5D/6D printing technology is gradually introduced into the food industry because it can produce more complex shapes with less materials. However, printability and printing efficiency limit the large-scale development of food printing technology. Currently, the methods to improve printability and printing efficiency mainly include changing the physiochemical properties of printing inks, optimizing printing settings, and upgrading printer design. The method to improve the printing printability of different food materials needs to consider the personalized purpose of printing products. The integrated printing equipment including multiple nozzles, post-processing, and intelligent detection are ideal preferences to realize commercial printing. Additionally, algorithm simulation represented by mathematical models, and instrument measurement represented by rheometer, low field nuclear magnetic resonance technology, and infrared spectroscopy, etc., are new methods to quickly evaluate the printability of raw materials. The development and establishment of intelligent food materials; food safety coatings; efficient and intelligent printing equipment; and quality standards are needed to invest in more research in order to promote the commercial development of food printing technology.

## Figures and Tables

**Table 1:** Effect of filling angle and filling percentage on the product deformation (induced by dehydration) based on 4D printing (Reprinted with permission from He et al., [42]. © 2020, Elsevier Ltd., Amsterdam, The Netherlands) [42].

Infill Condition and Model Dimension	Initial Configuration	Final Configuration
Infill angle: 15° Infill percentage: 60% Model size: 50 × 30 × 2.4 mm	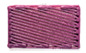	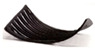
Infill angle: 45° Infill percentage: 60% Model size: 50 × 30 × 2.4 mm	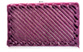	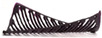
Infill angle: 75° Infill percentage: 60% Model size: 50 × 30 × 2.4 mm	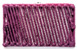	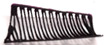
Infill angle: 90° (part 1); 0° (part 2) Infill percentage: 60% (parts 1 and 2); 100% (part 3) Model size: 70 × 70 × 2.4 mm	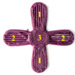	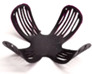
Infill angle: 0° (part 1); 60° (part 2); 120° (part 3) Infill percentage: 60% (parts 1, 2 and 3); 100% (part 4) Model size: 65 × 57 × 2.4 mm	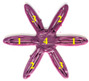	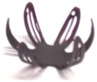
Infill angle: 0° (part 1 and 2) Infill percentage: 60% (parts 1 and 2); 100% (part 3) Model size: 72 × 54 × 2.4 mm	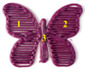	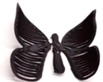
Infill angle: 45° (part 1); 135° (part 2) Infill percentage: 60% (parts 1 and 2); 100% (part 3) Model size: 72 × 54 × 2.4 mm	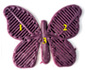	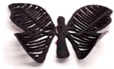

## Data Availability

Not applicable.
